# Perception of the Progressing Digitization and Transformation of the German Health Care System Among Experts and the Public: Mixed Methods Study

**DOI:** 10.2196/14689

**Published:** 2019-10-28

**Authors:** Arne Hansen, Maximilian Herrmann, Jan P Ehlers, Thomas Mondritzki, Kai Oliver Hensel, Hubert Truebel, Philip Boehme

**Affiliations:** 1 Didactics and Educational Research in Health Science Faculty of Health Witten/Herdecke University Witten Germany; 2 Johnson & Johnson Medical GmbH Production Planning & Logistics Norderstedt Germany; 3 Cardiovascular Research Bayer Aktiengesellschaft Wuppertal Germany; 4 Addenbrooke's Hospital Cambridge University Hospitals NHS Foundation Trust Cambridge United Kingdom; 5 Center for Clinical and Translational Research Department of Pediatrics Witten/Herdecke University Witten Germany

**Keywords:** digitization, health care sector, transformation, mixed method, delivery of health care, diffusion of innovation, reform

## Abstract

**Background:**

Health care systems worldwide are struggling to keep rising costs at bay with only modest outcome improvement among many diseases. Digitization with technologies like Artificial Intelligence or Machine Learning algorithms might address this. Although digital technologies have been successfully applied in clinical studies the effect on the overall health care system so far was limited. The regulatory ecosystem or data privacy might be responsible, but other reasons may also predominate.

**Objective:**

We analyzed how the digitization of the German health care market is currently perceived among different stakeholders and investigated reasons for its slow adaption.

**Methods:**

This was a mixed methods study split into a qualitative Part A using the conceptual approach of the Grounded Theory and a quantitative Part B using the Delphi method. For Part A we interviewed experts in the health care system and converted the results into 17 hypotheses. The Delphi method consisted of an online survey which was sent to the participants via email and was available for three months. For the assessment of the 17 hypotheses, the participants were given a six-point Likert scale. The participants were grouped into patients, physicians, and providers of services within the German health care market.

**Results:**

There was a strong alignment of opinions on the hypotheses between experts (N=21) and survey participants (N=733), with 70.5% overall agreement on 12/17 hypotheses. Physicians demonstrated the lowest level of agreement with the expert panel at 88% (15/17) disagreement, with the hypotheses “H8: Digitization in the health care system will free up jobs,” and “H6: Digitization in the health care system will empower the patients,” perceived to be in profound disagreement (*P*=.036 and *P*<.001, respectively).

**Conclusions:**

Despite the firm agreement among participants and experts regarding the impact of digitization on the health care system, physicians demonstrated a more negative attitude. We assume that this might be a factor contributing to the slow adoption of digitization in practice. Physicians might be struggling with changing power structures, so future measures to transform the market should involve them to a larger degree.

## Introduction

Health care systems worldwide are struggling, with aging societies and the western lifestyle leading to increasing health care expenditures [1-3]. Outcome-based reimbursement models have so far not gained the expected traction in the markets to compensate for higher levels of spending [4]. Particularly in the case of chronic conditions such as heart failure, chronic respiratory conditions, or diabetes, both hospitalizations and the continuum of care remain major cost drivers [5]. One problem is that innovations in the past were mainly based on medical therapy and inpatient treatment of acute diseases [6]. However, research has shown that many chronic condition outcomes can be improved by a lifestyle change and therapy adherence [7].

Several studies have shown the positive effects of technologies and digitalization in improving patient outcomes. For example, Schmier et al and Givertz et al demonstrated reduced hospitalization times and a reduction in costs using a remote monitor (CardioMEMS) for telemetric guided treatment of heart failure patients [8,9]. Furthermore, telehealth and telemedicine applications have demonstrated that they fill gaps, such as in the treatment of patients living in rural areas [10,11]. In some instances, higher therapy adherence induced by wearable sensors and mobile devices has been demonstrated [12-15].

As early as 2010, the Obama Administration set the mark with the Affordable Care Act (ACA) with the aim of transforming the health care system; for example, electronic health records [16]. Although the ACA focuses on technology, it remains unclear why many technologies have still not been implemented in clinical routine [17].

**Figure 1:**
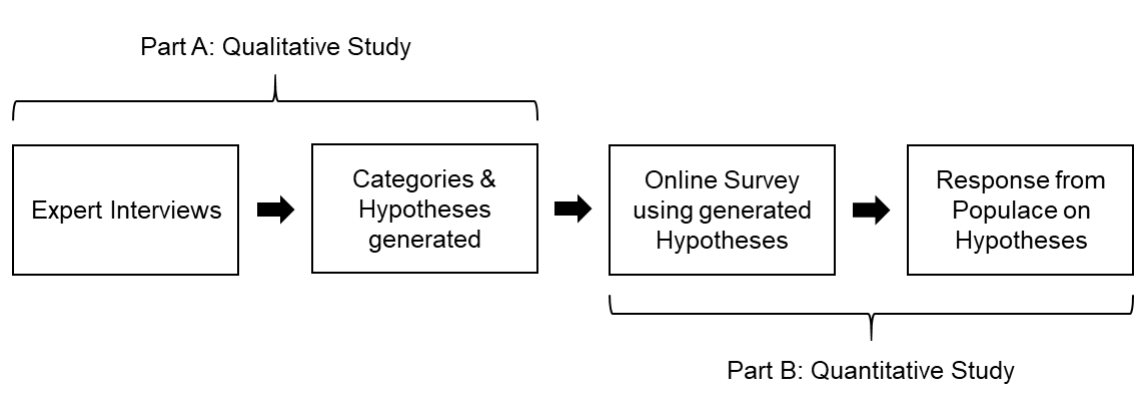
Flowchart study design.

This raises the question of whether the slow adoption of digital technologies might be caused by a lack of understanding of the benefits of these new technologies between the innovating experts who create them and the practitioners who will use them. Based on this question we conducted a two-stage mixed methods study, as seen in Figure 1.

In Part A, innovating experts (senior leaders) from different sectors within the health care market were interviewed to generate hypotheses on various aspects of the digitization of the health care system and any potential hurdles for the implementation of various technologies. In part B we performed a survey with more than 600 participants, differentiating between stakeholders in the health care system such as physicians, patients, and service providers.

## Methods

### Overview

This study was approved by the ethics committee of Witten/Herdecke University, Faculty of Medicine (application No. 169/2016), and conducted using an instrument development model which was divided into Part A and Part B, as described by Schifferdecker [18]. Part A was a qualitative study consisting of the conceptual approach of the Grounded Theory developed by Glaser and Strauss [19-21]. Interviews were done via telephone or face-to-face using an open interview guide (see Multimedia Appendix 1). The recorded responses were anonymous and were qualitatively evaluated using MAXQDA 13 (VERBI Software GmbH, Berlin, Germany) until saturation of hypotheses was reached. Part B represents a quantitative study using an online survey, based on the Delphi method, to test the generated hypotheses on the general public [22]. The results were then analyzed using MiniTab, version 17.1.0 (Minitab GmbH, Munich, Germany).

### Recruitment of Participants

For Part A, expert participants were chosen using the criteria of their job position in the German health care system. According to Glaser and Strauss, a heterogenic sample of participants is recommended to maximize the variations of experience within the group [19]. Therefore, we approached as many different healthcare experts that were concerned with innovation as possible to be participants in our study, to ensure a wide variety of insights for defining hypotheses. We also included senior executives and other senior stakeholders who were focused or had knowledge of the German health care system.

For Part B, surveyed participants from academia (eg, university medical centers) and private hospitals were asked to fill out four questions regarding their demographic background (age group, role in the health care system, job position, and country). We further reached out to patients and service providers (eg, employees of insurance companies). A six-point Likert scale ranging from one to six was used to assess each hypothesis.

### Data Collection

Part A data was collected from February 2017 to May 2017 via telephone or face-to-face interviews. Each interview was audio recorded with the permission of the participants. For Part B, the survey was published on SoGoSurvey and data was collected from November 2017 to February 2018.

The participants of this mixed methods study were informed upfront about data storage, the scope of the study, and the interviewer.

### Data Analysis

To assess the impact and influence of digitization on the German health care system and to obtain a general perception about it, an open interview guide was developed based on literature research and an expert panel consisting of members with multi-professional backgrounds in medicine, pharmaceuticals, and economics. Before using the interview guide for the study, it was tested in five pilot interviews, reviewed, and revised by the expert panel. The coding was performed based on the conceptual approach of the Grounded Theory [19] and the process was supported by literature research. The Grounded Theory approach was designed in three stages, starting with open coding, followed by axial coding, and then selective coding. The process was documented in memos to capture the progress and the ideas that emerged while creating the conceptual approach and analyzing the data [19-21]. For the analysis of the interviews, MAXQDA®13 was used to perform comparative data analysis of the quantitative data by two examiners. The interviews were coded without transcription. Further, the derived hypotheses were grouped into categories afterward to further distinguish the impact field of the different hypotheses. Values of the responses from the survey participants higher than 3.5 were considered to be in agreement with the experts, whereas values lower than 3.5 were considered to be in disagreement.

### Statistical Analysis

The data from the Delphi study was analyzed using MiniTab, version 17.1.0. For the calculation of the *P* value, a one-way analysis of variance (ANOVA) was applied under the assumptions of unequal variances and a statistical significance of *P*<.05.

## Results

### Part A: Expert Interview Results

#### Summary

**Figure 2:**
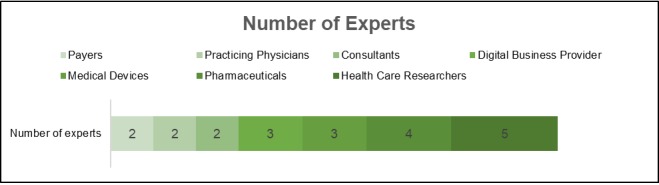
Number of experts.

In total, 30 experts were identified for this study. The interviews were conducted subsequently and stopped when saturation for the hypotheses was reached (N=21). The background of the experts is shown in Multimedia Appendix 2. Health Care Researchers represented the largest group, followed by pharmaceutical industry senior executives (Figure 2).

The experts responded to a total of nine questions in the interview guide (Multimedia Appendix 1). Based on these answers, 17 hypotheses (Table 1) were classified, which resulted in four categories (please see appendices for a full illustration of all hypotheses).

**Table 1:** List of hypotheses.

Number	Hypothesis
H1	Digitization will enable a disruptive structural change in the health care system.
H2	Key stakeholders in the health care system slow down digitization on purpose.
H3	Digitization in the health care system will improve the medical treatment of patients.
H4	Digitization in the health care system will bring more benefits to those with compulsory health insurance.
H5	Digitization in the health care system will establish new and homogenous communication structures which will increase transparency.
H6	Digitization in the health care system will empower the patients and change the power structures.
H7	Digitization in the health care system will increase self-monitoring and treatment of patients using digital devices.
H8	Digitization in the health care system will free up jobs and replace them with artificial intelligence, robots, etc.
H9	Digitization in the health care system will force pharmaceutical companies to develop products beyond the pill, (eg, hybrid models with additional service, or other applications or services) and further offer precision medicine.
H10	Big Data analysis of medical data, eg, interfacing between different professions will reduce malpractice and improve coordination of therapies.
H11	Digitization will secure medical care in underserved areas (eg, remote and rural areas).
H12	Digitization will increase the networking of stakeholders (eg, Physicians, Hospitals, Insurance and Pharmaceutical companies) within the health care system.
H13	Digitization will push the specialization of stakeholders (eg, Physicians, Hospitals, Insurance and Pharmaceutical companies) within the health care system.
H14	Digitization will offer opportunities to better differentiate caretakers from their competitors.
H15	Digitization in the health care system cannot replace the personal contact between stakeholders, such as between physicians or nurses and their patients.
H16	Digitization in the health care system will change existing job profiles.
H17	Digitization in the health care system leads to a depreciation of expert knowledge.

#### System

This category refers to hypotheses which influence the health care system in a holistic manner, such as, “H1. Digitization will enable a disruptive structural change in the health care system,” and about problems and opportunities caused by digitization, including, “H2: Key stakeholders in the health care system slow down digitization on purpose”. This category is the largest, with eight hypotheses in total.

#### Physician-Patient Relationship

This category covers all hypotheses dealing with the interaction of physicians and patients which will be significantly impacted by digitization, including, “H6: Digitization in the health care system will empower the patients and change the power structure,” and “H15: Digitization in the health care system cannot replace the personal contact between stakeholders, such as between physicians or nurses and their patients”. Three hypotheses were classified under this category.

#### Technology

This category represents all hypotheses which imply changes caused by digitization due to the availability of new technologies. Within the study, four hypotheses were identified for this category, such as, “H8: Digitization in the health care system will free up jobs and replace them by artificial intelligence,” and “H7: Digitization in the health care system will increase self-monitoring and treatment of patients using digital devices.”

#### Industry

This category deals with hypotheses which imply changes caused by digitization that will affect companies in the health care system and their provided services, including, “H9: Digitization in the health care system will force pharmaceutical companies to develop products beyond the pill (eg, hybrid models with additional services, or other applications or services) and further offer precision medicine,” and “H14: Digitization will offer opportunities to better differentiate caretakers from their competitors”. Two hypotheses are linked to this category.

### Part B: Delphi Study Results

#### Summary

**Figure 3:**
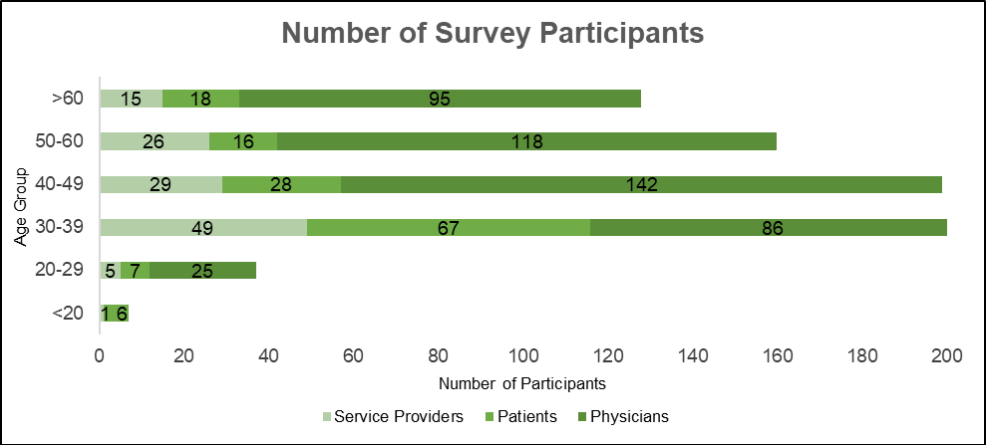
Number of survey participants.

A response rate of 20.9% (733/3500) was achieved. Among all surveyed participants, 1% (7/733) were under the age of 20, 5% (37/733) were between 20-29 years old, 27.5% (202/733) were between 30-39 years old, 27.1% (199/733) were between 40-49 years old, 21.8% (160/733) were between 50-60 years old, and 17.4% (128/733) were over 60 years old. A total of 63.5% (466/733) were physicians, 19.4% (142/733) were patients, and 17.1% (125/733) were service providers. Figure 3 shows that the largest group overall was represented by physicians between the ages of 40-49, with 19.3% (142/733). For service providers (6.68% [49/733]) and patients (9.14% [67/733]), their largest age group was 30-39 years old.

**Figure 4:**
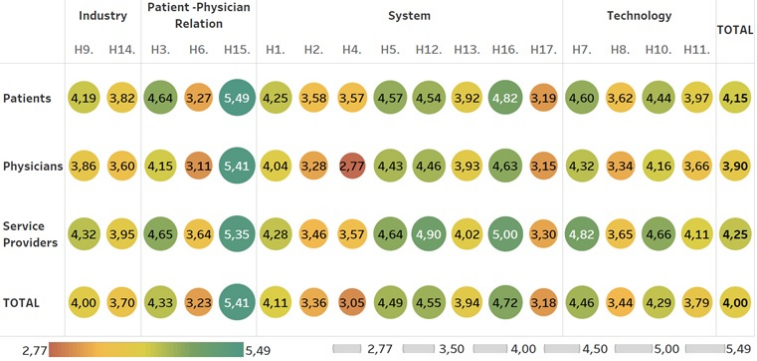
Level of agreement.

Overall, there was a 70.5% (12/17) agreement between the survey participants and the experts regarding changes in the health care system in Germany caused by digitization. Figure 4 shows the level of agreement using the mean from the survey participants regarding the hypotheses generated by the experts.

#### System

Among the System category, all survey participants agreed on 62.5% (5/8) of the hypotheses. Service providers showed the highest agreement, with a mean of 4.15 on the 6-point Likert Scale compared to patients (mean 4.06) and physicians (mean 3.84) (see Multimedia Appendix 3). The hypothesis, “H16: Digitization in the health care system will change existing job profiles,” had the highest approval, with a mean of 4.81 among survey participants. Regarding “H2: Key stakeholders in the health care system slow down digitization on purpose,” only patients agreed with it, with a mean of 3.58. In addition, for “H4: Digitization in the health care system will bring more benefits for those with compulsory health insurance,” only service providers and patients agreed, with a mean of 3.57 for both. However, the physicians demonstrated disagreement with this hypothesis with a mean of 2.77, which also represents the lowest mean for all 17 hypotheses. Moreover, the response on “H17: Digitization in the health care system leads to a depreciation of expert knowledge,” was the lowest in the category, with a mean of 3.18 among the survey participants. For this hypothesis, the physicians had the lowest mean of 3.15. H2 (*P*=.05), H4 (*P*<.001), H12 (*P*<.001) and H16 (*P*=.003) showed statistically significant differences (see Multimedia Appendix 1).

#### Physician-Patient Relationship

In total, the survey participants agreed on 66% (2/3) of the hypotheses regarding the Physician-Patient Relationship. Overall, the service providers demonstrated firm agreement, with a mean of 4.55 for this category compared to patients (mean 4.47) and physicians (mean 4.22). Concerning, “H15: Digitization in the health care system cannot replace the personal contact between stakeholders, such as between physicians or nurses and their patients,” this hypothesis had the highest mean of all 17 hypotheses (5.41) (see Multimedia Appendix 3). Regarding, “H6: Digitization in the health care system will empower the patients and change the power structures,” only the service providers agreed with a mean of 3.64. However, patients (mean 3.27) and physicians (mean 3.11) both disagreed. Statistically significant differences among the survey participants were found in H3 (*P*<.001) and H6 (*P*<.001) (see Multimedia Appendix 3).

#### Technology

The results for the Technology category showed an agreement among the survey participants of 75% (3/4) for all hypotheses. In total, the service providers had the highest mean (4.31) in this category compared to patients (mean 4.16) and physicians (mean 3.87). The highest agreement was identified for, “H7: Digitization in the health care system will increase self-monitoring and treatment of patients using digital devices,” with a mean of 4.58 (see Multimedia Appendix 3). With regards to, “H8: Digitization in the health care system will free up jobs and replace them by artificial intelligence, robots, etc,” only the physicians disagreed (mean 3.34). Overall, H7 (*P*<.001) and H10 (*P*<.001) showed statistical significance between the survey participants, and further for H8 (*P*=.036) and H11 (*P*=.002) (see Multimedia Appendix 3).

#### Industry

Looking at the Industry category, the results showed an agreement between the survey participants and the experts towards both hypotheses. The level of agreement in this category is again lead by the service providers (mean 4.13) compared to patients (mean 4.01) and physicians (mean 3.73) (see Multimedia Appendix 3). The hypothesis with the highest agreement was, “H9: Digitization in the health care system will force pharmaceutical companies to develop products beyond the pill (eg, hybrid models with additional service or other applications or services) and further offer precision medicine,” with a mean of 4.12. Statistically significant differences were detected between the survey participants for H9 (*P*<.001) and H14 (*P*=.011).

The service providers demonstrated the strongest agreement with the hypotheses, with the highest mean of 88% (15/17). In contrast, the physicians had the least agreement with the hypotheses, with a mean of 88% (15/17). Further, every category had one hypothesis where the survey participants were not aligned (ie, “H2: Key stakeholders in the health care system slow down digitization on purpose,” “H4: Digitization in the health care system will bring more benefits to those with compulsory health insurance,” “H6: Digitization in the health care system will empower the patients and change the power structures,” and “H8: Digitization in the health care system will free up jobs and replace them by artificial intelligence, robots, etc“). The physicians particularly disagreed on all four hypotheses. Moreover, all three groups disagreed with hypothesis, “H17: Digitization in the health care system leads to a depreciation of expert knowledge,” with a mean of 3.18 (see Multimedia Appendix 3). Overall, the results show an average mean of 4.25 for service providers, 4.15 for patients and 3.90 for physicians (see Multimedia Appendix 3).

## Discussion

### Key Findings

The results of this study demonstrate an overall limited impact and influence of digitization on the German health care system, based on the perception of the different participating groups. We found great agreement but also areas of incongruity between the various groups.

As Figure 4 shows, the survey participants agreed on the majority of the 17 hypotheses, although to different degrees. There particularly seemed to be a misalignment of opinions between the physicians and the experts. Compared to the service providers with a mean of 4.25, and patients with a mean of 4.15, the physicians had the lowest mean of 3.90 (see Multimedia Appendix 3). Interestingly, in a large study conducted by the US Physician Foundation in 2018, half of the respondents demonstrated a pessimistic attitude about the future [23]. This pessimistic view could be at least one reason the physicians had such a low mean. With respect to the category system, we observed interesting results related to the slow adaption of digitization. Patients agree with the experts from our panel, that main stakeholders are blocking a faster implementation, but physicians and service providers are significantly different and disagree. Nevertheless, if we look at Germany, the reluctance to implement a national electronic health record system due to data privacy issues raised by the German Government postponed this development significantly [24]. Further, the lowest level of agreement within our study was between experts and physicians. This result could indicate that physicians are particularly slowing down digitization, as this group will be among the most impacted in practice. Interestingly, the medical association slowed down the implementation of both telemedicine and the electronic health record in Germany [24,25].

We found great alignment between the survey participants and experts regarding the benefits associated with new technologies. The broad agreement regarding an increase of self-monitoring is one example showing how well digital devices are accepted. As Roess et al stated, more than 1200 mobile Health (mHealth) tools or apps are available that help patients obtain information and monitor their health status [26].

Apart from the technological advantages, the survey participants were not aligned on who would benefit the most from digitization. Although service providers and patients showed agreement with the experts that compulsory insured patients will benefit, physicians significantly disagreed. This could be based on German specifications, since as of 2018 there are 72.8 million people compulsory insured compared to 8.75 million with private health insurance in Germany [27]. Here, physicians have an incentive to treat privately insured patients and especially to provide them with treatments which are not covered by public insurance. Thus, these new forms of treatment often reach only a minority of patients. In comparison to other health care systems, characterized by generally high out-of-the-pocket payments, this might slow down innovation [28].

The relationship between physicians and patients is a very sensitive topic. Experts and survey participants both agreed that technology cannot replace personal contact, however, this data contrasts preexisting research. There are several studies which have demonstrated the benefits of robot applications for nursing and social interaction among older adults, and the high satisfaction of patients who used telemedicine visits or chat-bots [29-31]. In contrast, 78.7% of the physicians who participated in the US Physician Foundation survey pointed out that working with patients is the most satisfying factor in medical practice [23].

In addition to the potential loss of personal interaction, we found misalignment of opinions regarding patient empowerment. According to Topol et al, the introduction of smartphones and applications will empower patients, since they will be able to control all their relevant health care data on one device [32,33]. However, the data showed significant differences between the survey participants, with patients and physicians especially not supporting this hypothesis. This could be explained by a lack of understanding of the opportunities on behalf of the patients and a potential negative attitude among the physicians. There are major initiatives aiming at patient empowerment, eg, Patients Empowerment Campaign from the European Patients Forum and the Patient empowerment and health care guidelines from the World Health Organization. Both define processes and activities to enable patients to have greater control over decision making and consequential actions related to their health [34,35].

We also found disagreement among the survey participants regarding the depreciation of expert knowledge. Physicians seemed especially concerned, since they disagreed with the hypotheses about the replacements of jobs using AI applications. On the one hand, the physicians agreed with the results of recent studies which have already demonstrated technological potential for improved diagnostics and surgical decision making [36,37]. However, the responses indicate an underlying negative attitude among physicians regarding digitization when technology is no longer supporting but instead limiting or replacing their activities. This is problematic in two ways: (1) they could be detached from technological progress, with the threat being replaced in some areas; and (2) they are missing the opportunity to actively participate and enhance these new technologies with their experiences to achieve higher quality standards for their patients.

### Limitations

This study was performed within the German health care system with experts in Germany. While patient needs are comparable to other western societies, specific aspects in Germany (eg, a diverse payer and provider landscape compared to the United Kingdom) might limit its applicability to other systems. Therefore, the implications derived from this study concerning the common understanding of the impacts and influences of digitization might not be applicable to other health care systems in the world.

### Conclusion

The digitization of the health care sector in Germany could cause significant changes, and only the future will tell how different stakeholders will be able to adapt. According to our research, the current adaptation level varies strongly among different participants in the market. For some of them we found significant alignment of opinions between experts and survey participants (eg, referring improved medical treatment, standardized communication structures, increased self-monitoring of patients and the importance of the personal contact between patient and physician in a digitized relationship). However, substantial agreement gaps exist regarding the empowerment of patients, the application of artificial intelligence and robots and thus the replacement of expert knowledge, particularly between physicians and our expert panel.

Physicians showed a negative attitude towards the empowerment of patients that comes with the process of digitization. They also failed to recognize that, in some areas, they might be replaced by technology. To generate the highest value for patients and to bring the technological advances to patients as fast as possible, it is crucial to involve all stakeholders. This is especially important in cases where job profiles will change. Physicians should acknowledge the change introduced by technological transformation and play a more active and positive role.

## Appendix

Multimedia Appendix 1 Open Interview Guide.

Multimedia Appendix 2 Job Profile & Industry Sector Expert Panel.

Multimedia Appendix 3 Overview Results Delphi Method Survey.

## Abbreviations

ACA: Affordable Care Act
ANOVA: analysis of variance
mHealth: mobile Health
