# Supplementary material for: Perception of the Progressing Digitization and Transformation of the German Health Care System Among Experts and the Public: Mixed Methods Study
Source: JMIR Public Health Surveill. 2019 Oct 28;5(4):e14689. doi: 10.2196/14689 (PMC6913772; doi:10.2196/14689)
Supplement: Multimedia Appendix 1 [file publichealth_v5i4e14689_app1.pdf]

Number	Question
1.	What is the influence of the increasing digitization on the German and international health care system?
2.	How will this change our health care system?
3.	How will the staffing ratio in the health care system change in the course of the digitization?
4.	Does the digitization lead to an allocation or rather a shortage of resources for private or compulsory insured persons?
5.	How will the different value chains in the health care system change? Keyword = Democratization?
6.	How will the digitization change on the medical treatment of patients?
7.	How will the business model of large pharmaceutical companies change?
8.	Will the digitization ensure medical security of supply in rural areas?
9.	What does digitization mean to you personally?
