# Supplementary material for: Perception of the Progressing Digitization and Transformation of the German Health Care System Among Experts and the Public: Mixed Methods Study
Source: JMIR Public Health Surveill. 2019 Oct 28;5(4):e14689. doi: 10.2196/14689 (PMC6913772; doi:10.2196/14689)
Supplement: Multimedia Appendix 2 [file publichealth_v5i4e14689_app2.pdf]

Job Profile Expert	Industry Sector
President University / Physician	Health Care Researcher
Professor Innovation Health Care System	Health Care Researcher
Author / Foundation lead	Health Care Researcher
Professor Social Research	Health Care Researcher
State Doctorate / Head of Clinical Science	Health Care Researcher
Head of Innovation	Pharmaceuticals
Business Unit Lead	Pharmaceuticals
General Manager	Pharmaceuticals
Market Access Lead	Pharmaceuticals
General Manager Germany	Medical Devices
Medical Director DACH	Medical Devices
General Manager Strategy	Medical Devices
Global Head / SVP	Digital Business Provider
Social Impact Lead & Global Partnerships	Digital Business Provider
CEO	Digital Business Provider
Health Care Market Expert	Consultant
Physician / Health Care Market Expert	Consultant
Internal Medicine Specialist/Medical Center CEO	Practicing Physicians
President Medical Association / Physician	Practicing Physicians
Digital Office Lead	Payer
Head of Department “Digitalization Market”	Payer
