# Supplementary material for: Perception of the Progressing Digitization and Transformation of the German Health Care System Among Experts and the Public: Mixed Methods Study
Source: JMIR Public Health Surveill. 2019 Oct 28;5(4):e14689. doi: 10.2196/14689 (PMC6913772; doi:10.2196/14689)
Supplement: Multimedia Appendix 3 [file publichealth_v5i4e14689_app3.pdf]

Hypotheses - System	Group	Mean	P-Value
**H1. Digitization will enable a disruptive structural change in the health care system.**	Service Providers	**4,28**	0.08
	Patients	4,25	
	Physicians	4,04	
**H2. Key stakeholders in the health care system slow down digitization on purpose**	Service Providers	3,46	0.05*
	Patients	**3,58**	
	Physicians	3,28	
**H4. Digitization in the health care system will bring more benefits for those with compulsory health insurance.**	Service Providers	**3,57**	<0.001*
	Patients	**3,57**	
	Physicians	2,77	
**H5. Digitization in the health care system will establish new and homogenous communication structures which will increase transparency.**	Service Providers	**4,64**	0.231
	Patients	4,57	
	Physicians	4,43	
**H12. Digitization will increase the networking of the stakeholders (eg, Physicians, Hospitals, Insurance and Pharmaceutical companies) within the health care system.**	Service Providers	**4,90**	<0.001*
	Patients	4,54	
	Physicians	4,46	
**H13. Digitization will push the specialization of stakeholders (eg, Physicians, Hospitals, Insurance and Pharmaceutical companies) within the health care system.**	Service Providers	**4,02**	0.768
	Patients	3,92	
	Physicians	3,93	
**H16. Digitization in the health care system will change existing job profiles.**	Service Providers	**5,00**	0.003*
	Patients	4,82	
	Physicians	4,63	
**H17. Digitization in the health care system leads to a depreciation of expert knowledge.**	Service Providers	**3,30**	0.605
	Patients	3,19	
	Physicians	3,15	

Hypotheses - Patient -Physician Relation	Group	Mean	P-Value
**H3. Digitization in the health care system will improve the medical treatment of patients.**	Service Providers	**4,65**	<0.001*
	Patients	4,64	
	Physicians	4,15	
**H6. Digitization in the health care system will empower the patients and change the power structures.**	Service Providers	**3,64**	<0.001*
	Patients	3,27	
	Physicians	3,11	
**H15. Digitization in the health care system cannot replace the personal contact between stakeholders, such as between physicians or nurses with their patients.**	Service Providers	5,35	0.573
	Patients	**5,49**	
	Physicians	5,41	

Hypotheses - Technology	Group	Mean	P-Value
**H7. Digitization in the health care system will increase self-monitoring and treatment of the patients using digital devices.**	Service Providers	**4,82**	<0.001*
	Patients	4,60	
	Physicians	4,32	
**H8. Digitization in the health care system will free up jobs and replace them by artificial intelligence, robots etc..**	Service Providers	**3,65**	0.036*
	Patients	3,62	
	Physicians	3,34	
**H10. Big Data analysis of medical data, eg, interfacing between different professions will reduce malpractice and improve coordination of therapies.**	Service Providers	**4,66**	<0.001*
	Patients	4,44	
	Physicians	4,16	
**H11. Digitization will secure the medical care in underserved areas (eg, remote and rural areas).**	Service Providers	**4,11**	0.002*
	Patients	3,97	
	Physicians	3,66	

Hypotheses - Industry	Group	Mean	P-Value
**H9. Digitization in the health care system will force pharmaceutical companies to develop products beyond the pill, (eg, hybrid models with additional service, or other applications or services) and further offer precision medicine.**	Service Providers	**4,32**	<0.001*
	Patients	4,19	
	Physicians	3,86	
**H14.Digitization will offer opportunities to better differentiate caretakers from their competitors**	Service Providers	**3,95**	0.011*
	Patients	3,82	
	Physicians	3,60	
